# The Expression of lncRNA NEAT1 in Human Tuberculosis and Its Antituberculosis Effect

**DOI:** 10.1155/2018/9529072

**Published:** 2018-11-11

**Authors:** Shuying Huang, Zikun Huang, Qing Luo, Cheng Qing

**Affiliations:** ^1^Department of Obstetrics and Gynecology, The First Affiliated Hospital of Nanchang University, Nanchang, 330006 Jiangxi, China; ^2^Department of Nursing, Jiangxi Health Vocational College, Nanchang, 330052 Jiangxi, China; ^3^Department of Clinical Laboratory, The First Affiliated Hospital of Nanchang University, Nanchang, 330006 Jiangxi, China; ^4^Intensive Care Unit, The First Affiliated Hospital of Nanchang University, Nanchang, 330006 Jiangxi, China

## Abstract

Increasing evidence suggests that lncRNA is important in innate immune responses. Recent study has demonstrated that lncRNA NEAT1 (which has two subtypes: NEAT1_1 and NEAT1_2) nuclear-enriched abundant transcript 1 (NEAT1) is essential in immune regulation, but the expression and clinical significance in tuberculosis are still unclear. In this work, we aimed to discuss the expression and clinical significance of NEAT1 in tuberculosis patients. Quantitative real-time polymerase chain reaction was performed to detect the expression of NEAT1 (both NEAT1_1 and NEAT1_2) in peripheral blood mononuclear cells (PBMCs) of patients with tuberculosis and healthy controls and analyze the association of NEAT1 with the development, progression, and outcome of tuberculosis. Then NEAT1 was silenced in THP-1 cells using siRNA. The expression of tumor necrosis factor- (TNF-) *α* and interleukin- (IL-) 6 was detected after Mycobacterium tuberculosis (Mtb) infection, and the change in bactericidal capacity against Mtb was assessed. We demonstrated that the relative expression of NEAT1 (both NEAT1_1 and NEAT1_2) in patients with tuberculosis was higher than that in the control. However, the expression of NEAT1 (both NEAT1_1 and NEAT1_2) in the new case and relapse groups had insignificant differences. The level of NEAT1 (both NEAT1_1 and NEAT1_2) in PBMCs declined gradually with treatment and was restored to the normal level. The expression of NEAT1 (both NEAT1_1 and NEAT1_2) in THP-1 cells increased markedly after Mtb infection. The levels of IL-6 but not TNF-*α* in Mtb-infected THP-1 cells declined after the NEAT1 (both NEAT1_1 and NEAT1_2) knockout. The survival of Mtb in NEAT1-knockout (both NEAT1_1 and NEAT1_2) THP-1 cells reached its peak 72 h after infection, taking 0 h after Mtb infection as the baseline data; the difference was statistically significant compared with the control. Thus, our results indicate that the expression of NEAT1 increased during Mtb infection, and it might be associated with the outcome of tuberculosis. The decreased expression of NEAT1 might weaken the clearance of intracellular Mtb by macrophages.

## 1. Introduction

Tuberculosis is a chronic infectious disease significantly affecting human health. Recently, the treatment of tuberculosis has become even more difficult due to a continuous increase in the number of resistant strains, and the occurrence of extensively drug-resistant tuberculosis brings new challenges to antituberculosis treatment [1]. A long-term epidemiologic study has shown that, although the prevalence of Mycobacterium tuberculosis (Mtb) infection is high, only 5%–10% of the infections would develop into active tuberculosis, and the immune system is implicated in the development and progression of this disease [2]. Mtb is an intracellular parasite that lives in macrophages after infecting humans. The human body mainly relies on cellular immunity to fight against tuberculosis. However, how Mtb escapes from immune surveillance is still unclear, and hence tuberculosis is difficult to control.

Long noncoding RNA (lncRNA) were RNA molecules with a length of more than 200 nucleic acids. It is widely expressed in eukaryotes and participates in various biological processes such as cell proliferation, differentiation, apoptosis, and development [3]. Increasing evidence suggests that lncRNA is also involved in immune regulation, especially in innate immune responses [4]. For example, lncRNA Lethe [5] could inhibit the binding between nuclear factor kappaB (NF-*κ*B) and chromosomal DNA, thereby blocking inflammatory immune responses mediated by NF-*κ*B.

Paraspeckles are giga-dalton protein complexes which consists of a huge protein-protein interaction network (p54nrb, PSF, and PSPp1) hold together by the lncRNA NEAT1 with the three DBHS proteins as major key player [6, 7]. NEAT1 can interact with many intracellular regulatory factors and therefore is involved in the formation, differentiation, and metastasis of colon cancer [8], gallbladder cancer [9], ovarian cancer [10], prostate cancer [11], and many others. A recent study has demonstrated that lncRNA NEAT1, as a new regulatory molecule, which has two subtypes, NEAT1_1 (3.7 kb) and NEAT1_2 (23 kb), is essential in immune regulation [12, 13]. However, the expression of NEAT1 in patients with tuberculosis is still unclear because the research on lncRNA in tuberculosis is still in its initial stage.

In this study, real-time quantitative polymerase chain reaction (qRT-PCR) was performed to detect the expression of NEAT1 in peripheral blood mononuclear cells (PBMCs) of patients with tuberculosis and analyze the association of NEAT1 with the development, progression, and outcome of tuberculosis. In addition, an Mtb-infecting THP-1 human macrophage model was established to further analyze the in vitro effect of NEAT1 on Mtb infection, thereby highlighting the influence and significance of NEAT1 in tuberculosis.

## 2. Materials and Methods

### 2.1. Study Subjects

Tuberculosis group: this study included a total of 106 patients with tuberculosis who were hospitalized in the Department of Tuberculosis of Jiangxi Chest Hospital, China, from June 2014 to June 2015. Of these, 61 were male and 45 were female. The age of the patients ranged from 20 to 59 years, and the median age was 36 years; 75 were new cases, and 31 were relapse cases. According to clinical manifestations, bacteriologic detection, imaging examination, or history of antituberculosis treatment, all patients were correctly diagnosed with tuberculosis and typed as Mtb after Mtb genotyping.

Healthy control: the healthy control group included 55 subjects who underwent routine physical examination in the Department of Health Care of the First Affiliated Hospital of Nanchang University, China, during the same period. Of these, 31 were male and 24 were female; the age range was 21–61 years, and the median age was 39 years. No abnormalities were found on chest x-rays. The controls did not have recent contact with patients with tuberculosis and had negative results for the PPD test. All subjects were excluded from a combination of tumor, hypertension, diabetes, autoimmune diseases, or history of immunosuppressive drug intake. Pregnant and lactating women and patients with hepatitis B virus, hepatitis C virus, human immunodeficiency virus (HIV), and(or) acquired immunodeficiency syndrome were excluded. This study was approved by the ethics committee of Jiangxi Chest Hospital, China, and all patients gave their informed consent. The patients with tuberculosis and healthy controls had no significant difference in age or gender (P > 0.05).

### 2.2. Reagents and Instruments

THP-1 human macrophages were purchased from Shanghai Institute of Cell Research, Chinese Academy of Sciences; Mtb standard strain H37Rv (ATCC 27294) was preserved by the laboratory. The Ficoll lymphocyte separation medium, fetal bovine serum, and phorbol myristate acetate (PMA) were procured from Sigma (USA); the transfection reagent Lipofectmine 2000 and Trizol from Invitrogen (USA); the PrimeScript reverse transcription reagent kit with gDNA Eraser and SYBR Premix Ex Taq from TaKaRa (Dalian, China); DMEM medium from Hyclone (USA); the Opti-MEM I Reduced Serum Medium for transfection from Gibco (USA); the Middlebrook 7H10 medium and OADC from BD (USA); and the enzyme-linked immunosorbent assay (ELISA) kit detecting human TNF-*α* and IL-6 from eBioscience (USA). The spectrophotometer was purchased from Bio-Rad (USA), and the Applied Biosystems 7600 thermocycler from ABI (USA).

*PBMC Isolation. *Morning fasting venous blood was collected in a 5-mL tube with heparin sodium for anticoagulation. PBMCs were routinely isolated using the Ficoll lymphocyte separation medium.

### 2.3. THP-1 Cell Culture

THP-1 is a human-derived monocytic cell line that grows in suspension. THP-1 was routinely cultured in this study: the cell suspension was adjusted to a concentration of 1 × 10^6^ ml^−1^, inoculated in a 12-well plate (2 mL per well), and cultured with PMA (30 ng/mL) for 48 h. Cell attachment was observed: if cells were well attached and aggregated, it meant that they were differentiated into mature mononuclear macrophages.

### 2.4. Establishment of the Infection Model

Mature THP-1 cells were inoculated into a 24-well plate. After attachment, the cells were infected with Mtb at a series of multiplicity of infection (MOI), washed with phosphate-buffered saline (PBS) after 4 h to remove uninfected bacteria, cultured in an incubator at 37°C in the presence of 5% CO_2_, and extracted with RNA 12 h after infection. Or, the cells were infected with Mtb at an MOI of 5, washed with PBS after 4 h to remove uninfected bacteria, cultured in an incubator at 37°C and in the presence of 5% CO_2_, and extracted with RNA 3, 6, 12, 24, and 48 h after infection. The expression of NEAT1 was detected by qRT-PCR.

### 2.5. Design and Synthesis of siRNA

The siRNA for NEAT1_1, NEAT1_2, and control siRNA (siNC) were synthesized by Gene Pharma (Shanghai, China). The siRNA sequences [11] were as follows: NEAT1_1 siRNA1 (5′-UGGUAAUGGUGGAGGAAGAUU-3′), NEAT1_1 siRNA2 (5′-GUGAGAAGUUGCUUAGAAAUU-3′), and NEAT1_1 siRNA3 (5′-GGAGGAGUCAGGAGGAAUAUU-3′); NEAT1_2 siRNA1 (5′-CCAAAUAGGCUUACAGAUAUU-3′) and NEAT1_2 siRNA2 (5′-AGAGAGAAGUUGUGGAGAAUU-3′).

### 2.6. Cell Transfection

The cells were divided into blank control, siNC infection group, and si-NEAT1 infection group. Mature THP-1 cells were inoculated into a 24-well plate. After attachment the cells were transfected with si-NEAT1 or siNC according to the manufacturer's instruction provided with the Lipofectamine 2000 reagent. The transfected cells were then placed in an incubator, and the medium was changed after 4–6 h. The result of siRNA silencing was detected by qRT-PCR 24 h after transfection.

### 2.7. RNA Extraction and cDNA Synthesis

Total RNA was extracted and dissolved in distilled water treated with DEPC according to the manufacturer's instruction provided with TRIzol reagent. The concentration and purity of RNA were determined using a spectrophotometer, and qualified RNA was preserved at –80°C. For cDNA synthesis, 1 *μ*L of RNA was taken and reverse transcribed into cDNA using the PrimeScript RT Reagent Kit with gDNA Eraser.

### 2.8. qRT-PCR

qRT-PCR was performed using the SYBR Green method. All detection steps and reaction conditions were set according to the instruction provided with the kit. Glyceraldehyde-3-phosphate dehydrogenase (GAPDH) was used as internal reference [14].The sequences for NEAT1_1 primers were as follows [14]: upstream: 5′-CTTCCTCCCTTTAACTTATCCATTCAC-3′; downstream: 5′- CTCTTCCTCCACCATTACCAACAATAC-3′. The sequences for NEAT1_2 primers were as follows: upstream: 5′-CAGTTAGTTTATCAGTTCTCCCATCCA-3′ 5; downstream: ′-GTTGTTGTCGTC-ACCTTTCAACTCT-3′. The sequences for GAPDH primers were as follows: upstream: 5′-TGCACCACCAACTGCTTAGC-3′; downstream: 5′-_GGCATGGACTGTGGTCATGAG -3′. The 20-*μ*L reaction system included 1×SYBR Green I Master Mix, 0.5 *μ*mol/L forward primer, 0.5 *μ*mol/L reverse primer, and 1 *μ*L of reverse transcription product. The reaction conditions were set as follows: predenaturation at 95°C for 10 min, 95°C for 15 s, 60°C for 1 min, 72°C for 30 s, and 40 cycles. qRT-PCR was performed using the ABI 7600 thermocycler. All samples had three repeats. The result of qRT-PCR was analyzed according to the Ct value, taking GAPDH as the internal reference and using the relative quantitative method.

### 2.9. TNF-*α* and IL-6 Detection

NEAT1-silenced THP-1 macrophages were infected with Mtb at an MOI of 5, and the levels of TNF-*α* and IL-6 were detected by ELISA 48 h after infection.

### 2.10. Bactericidal Capacity of THP-1 against Mtb

NEAT1-silenced THP-1 macrophages were infected with Mtb at an MOI of 5. The cells were washed with PBS to remove uninfected bacteria 4 h later, and this time point was set as 0 h. Then, the cells were lysed with 0.05% SDS in saline at 0 and 72 h, and the lysate was serially 10-fold diluted with Hanks and spread on a 7H10 plate (containing OADC) for 3–4 weeks of culturing at 37°C. Then, the colonies were counted, and the number of bacteria in cells was measured.

### 2.11. Statistical Analysis

A statistical analysis was performed using SPSS 17.0 software (SPSS Inc., USA). Data with a normal distribution (evaluated using Kolmogorov–Smirnov and Shapiro–Wilk tests) were presented as mean ± standard deviation. The mean comparison of the two samples was performed using the t test, and multigroup comparison was performed using analysis of variance. A P value <0.05 was defined as statistical significance.

## 3. Results

### 3.1. NEAT1 Expression in PBMCs of Patients with Tuberculosis and Healthy Controls

**Figure 1:**
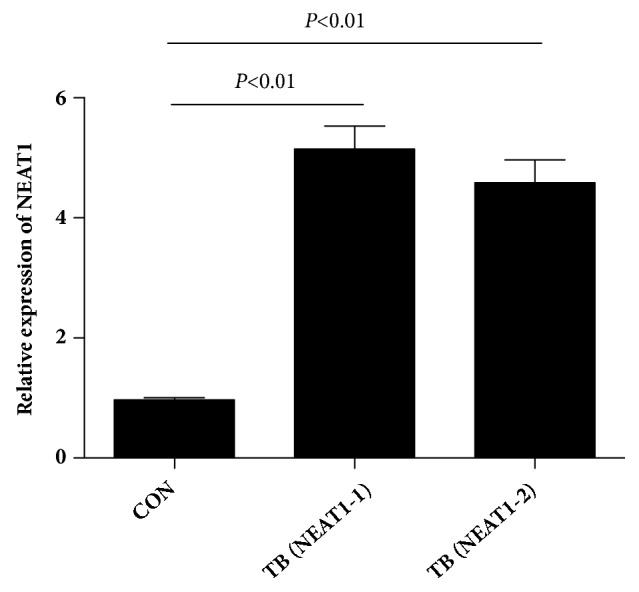
Relative expression of NEAT1_1 and NEAT1_2 in PBMC samples from patients with tuberculosis and healthy controls.

The expression of NEAT1 in PBMCs of 106 patients and 55 controls was detected by qRT-PCR. As shown in Figure 1, patients with tuberculosis had significantly higher expression of NEAT1_1 and NEAT1_2 in their PBMCs, and the level was 5.14 ± 0.89 and 4.58±0.83 times of the normal. The difference was statistically significant (P < 0.01).

### 3.2. Expression of NEAT1_1 and NEAT1_2 in PBMCs of Patients with New or Relapse Tuberculosis

**Figure 2:**
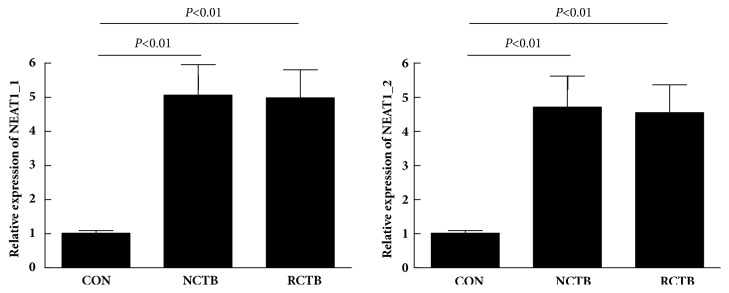
Relative expression of NEAT1_1 and NEAT1_2 in PBMC samples from different groups. NCTB, new cases of tuberculosis; RCTB, relapse cases of tuberculosis.

Patients were further divided into new a case group (n = 75) and a relapse case group (n = 31) according to the history of antituberculosis treatment. The analysis of the expression of NEAT1_1 and NEAT1_2 showed no significant difference between the two groups of patients (P > 0.05) (Figure 2).

### 3.3. Variation in the NEAT1_1 and NEAT1_2 Level in PBMCs of Patients with Tuberculosis Following Antituberculosis Treatment

**Figure 3:**
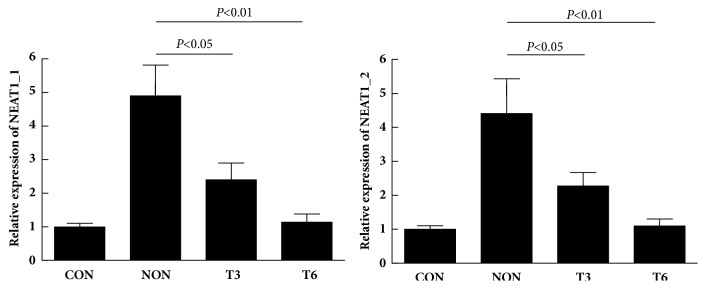
Relative expression of NEAT1_1 and NEAT1_2 in PBMC samples following effective anti-TB chemotherapy. NON, no treatment; T3, 3 months after treatment; T6, 6 months after treatment.

A 6-month follow-up was conducted on 42 new cases, and the variation in the NEAT1_1 and NEAT1_2 level in PBMCs was measured at 3 and 6 months after treatment to analyze whether dynamic changes in NEAT1_1 and NEAT1_2 could reflect the efficacy of antituberculosis treatment. The result showed that the effect of antituberculosis treatment was good. The expression of NEAT1_1 and NEAT1_2 decreased gradually with the increase in treatment time (Figure 3). Patients receiving treatment had the significantly lowered expression of NEAT1_1 and NEAT1_2 3 and 6 months after treatment compared with the untreated group (P < 0.05), and the NEAT1_1 and NEAT1_2 level of patients was insignificantly different from that of the healthy control after 6 months of treatment (P > 0.05).

### 3.4. Change of NEAT1_1 and NEAT1_2 in THP-1 Cells after Mtb Infection

**Figure 4:**
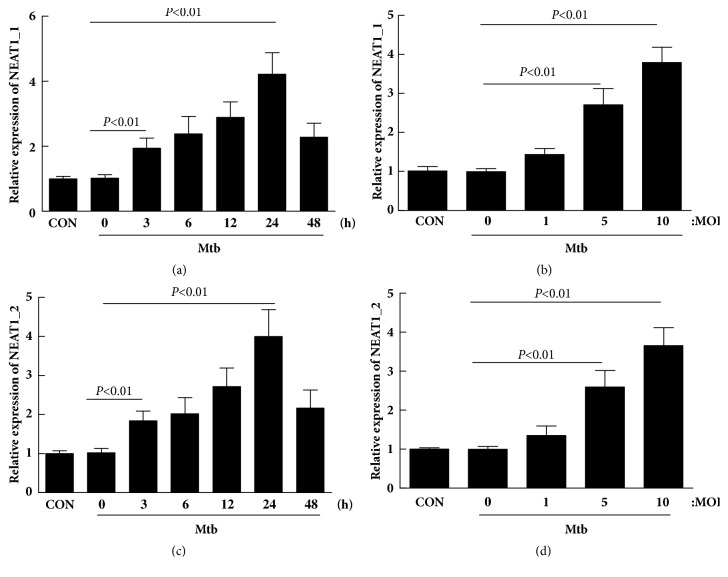
Mycobacterium tuberculosis infection robustly increased the expression of NEAT1_1 and NEAT1_2 in THP-1 macrophages in vitro.

Mtb was used to infect THP-1 cells at an MOI and for different times to analyze the influence of Mtb on the expression of NEAT1_1 and NEAT1_2. As shown in Figure 4, the expression of NEAT1_1 and NEAT1_2 increased 3 h after infection and reached the peak at 24 h (P < 0.01). In addition, the expression of NEAT1_1 and NEAT1_2 was also upregulated and reached the peak at MOI = 10 with the increase in MOI (P < 0.05).

### 3.5. Change in TNF-*α* and IL-6 Secretion after NEAT1 Silencing

**Figure 5:**
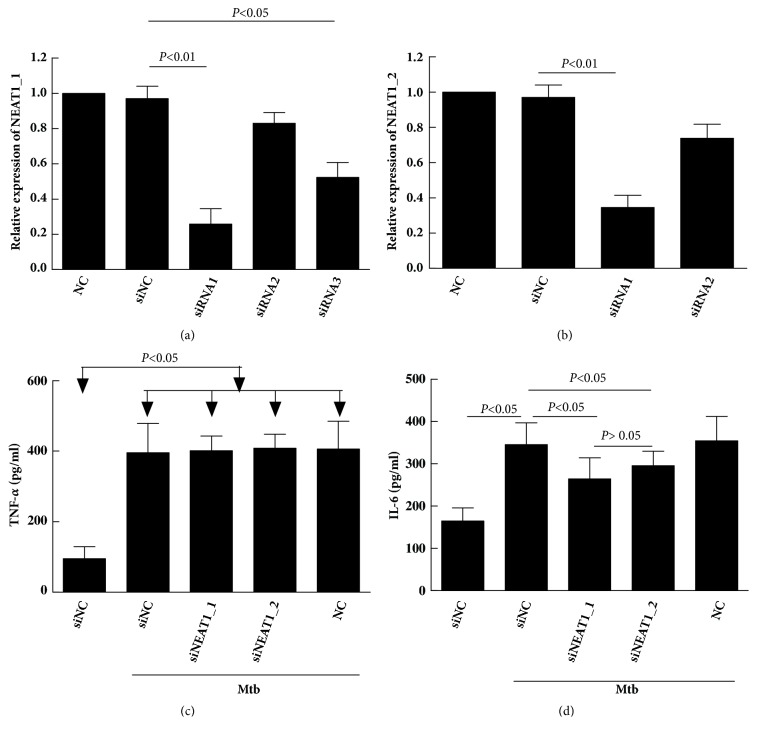
Differential secretory volume of TNF-*α* and IL-6 in THP-1 macrophages knocked down by NEAT1_1 and NEAT1_2 after Mycobacterium tuberculosis infection.

First step, NEAT1_1 and NEAT1_2 were silenced using siRNAs targeting at different sites. For NEAT1_1, the result of qRT-PCR detection showed that the transfection with siNEAT1-1 and siNEAT1-3 significantly lowered the expression of NEAT1, showing an inhibition rate of 74.2% and 47.7%, respectively, compared with the control (P < 0.05). Although siNEAT1-2 inhibited the expression of NEAT1 to a certain level, the difference was insignificant compared with the control (P > 0.05) (Figure 5(a)). siNEAT1-1 was used in the following experiments because it showed the highest interfering effect. For NEAT1_2, also the siNEAT1-1 showed a better inhibition rate (34.5% versus 73.7% ) which was used in the following experiments (Figure 5(b)). The second step, NEAT1_1 and NEAT1_2 knockout THP-1 cells were infected with Mtb, and the suspension was collected for ELISA detection of TNF-*α* and IL-6. As shown in Figures 5(c) and 5(d), the levels of TNF-*α* and IL-6 were extremely low in normal cells, but the secretion of the two cytokines increased on adding Mtb (*P *< 0.05). The cells with silenced NEAT1_1 and NEAT1_2 showed the decreased expression of IL-6 but not TNF-*α* compared with cells transfected with siNC, and the difference was statistically significant (*P* < 0.05). But there was no difference in IL-6 between NEAT1_1 and NEAT1_2 group (*P* >0.05).

### 3.6. Bactericidal Capacity of THP-1 against Mtb after NEAT1 Silencing

**Figure 6:**
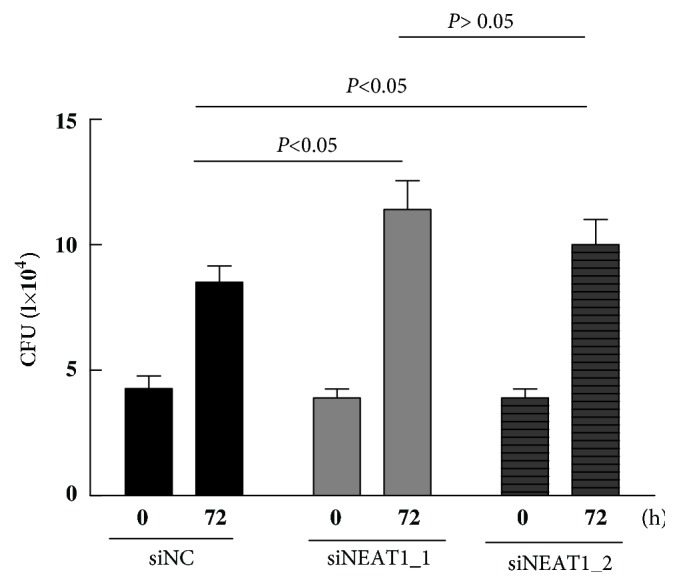
Effects of NEAT1 on the bactericidal capacity of THP-1 macrophages.

After NEAT1_1 and NEAT1_2 silencing, THP-1 cells were infected with Mtb, lysed, and cultured on a solid medium to measure the level of bacteria. The result showed that the number of bacteria in NEAT1_1 and NEAT1_2 silenced cells was insignificantly different from that in the control cells 0 h after Mtb infection (*P *> 0.05), indicating that the downregulation of NEAT1_1 and NEAT1_2 did not affect the phagocytic function of macrophages. Considering the level at 0 h as the baseline, it was found that the survival of Mtb in THP-1 cells treated with siNEAT1_1 and siNEAT1_2 was significantly higher than that in the control after 72 h of Mtb infection (Figure 6), implying that NEAT1_1 and NEAT1_2 downregulation might significantly inhibit the ability of macrophages to kill intracellular Mtb. But there was no difference in kill intracellular Mtb between NEAT1_1 and NEAT1_2 group (Figure 6).

## 4. Discussion

Increasing evidence suggests that lncRNA is important in the development and progression of many human diseases [15]. The regulatory effects of lncRNA in tuberculosis have also attracted widespread attention in recent years. For example, Yi et al. [16] used a microarray to detect CD4+ T cells in the peripheral blood and found that lncRNA showed abnormal expression in patients with active or latent tuberculosis compared with healthy controls. A recent study also confirmed that the T-cell inhibitory factor CD244 epigenetically controlled CD8+ T-cell immune responses during tuberculosis infection by inducing the expression of lncRNA CD244[17].

NEAT1 is also named multiple endocrine neoplasia (MEN)*ε*/*β* or VINC. It is transcribed by RNA polymerase II from MEN I gene loci located on human chromosome 11. Both NEAT1_1 and NEAT1_2 were detected in PBMCs of patients with tuberculosis in the present study.

Recent studies showed that NEAT1 was also actively involved in immunoregulation and had important functions in innate immune responses. Zhang et al. [18] showed that NEAT1 was upregulated in T cells infected by HIV-1, whereas NEAT1 knockout increased HIV-1 replication, and this was achieved by increasing the transport of HIV-1 mRNA from nucleus to cytoplasm. Hantavirus infection also increased the expression of NEAT1 in human umbilical vein endothelial cells. The upregulated NEAT1 induced IFN secretion, and the mechanism might be associated with the activation of RIG-I/IRF7 signaling pathway [19]. Zhang et al. [13] found that lipopolysaccharide stimulated the expression of NEAT1 in macrophages, and upregulated NEAT1 could affect the expression of cytokines via the JNK/ERK MAPK signaling pathway. Our previous study found that NEAT1_1 expression of PBMCs in sepsis and SIRS patients were significantly increased [20], which could be considered as a good additive marker for the diagnosis of sepsis, but the study excluded TB patients. So far, no study to date has reported the role of NEAT1 in tuberculosis.

qRT-PCR was performed in this study to detect the expression of NEAT1 in PBMCs of patients with tuberculosis and healthy controls. The results showed that NEAT1_1 and NEAT1_2 were significantly elevated in the tuberculosis group, whereas no significant difference was identified between the new case group and the relapse case group. In addition, some new cases were followed up for 6 months, and during this period, the patients were treated in strict accordance with the standard tuberculosis treatment program. No patient received radiotherapy, chemotherapy, hormonotherapy, or other therapies, and all patients had a good treatment outcome. Treated cases had significantly lowered the expression of NEAT1_1 and NEAT1_2 3 months later compared with the untreated patients. The level of NEAT1_1 and NEAT1_2 further decreased with the increase in treatment time and the improvement of disease and fell to a normal level insignificantly different from that in the healthy control after 6 months of treatment.

Studies show that Mtb lives mainly in macrophages after infecting humans. Therefore, possibly all actions against Mtb would finally be exerted by macrophages [20]. qRT-PCR was used in this study to detect the variation in the expression of NEAT1_1 and NEAT1_2 in human macrophage cell line THP-1so as to analyze the role of NEAT1 in Mtb infection. The result showed that cells with Mtb infection had significantly elevated the expression of NEAT1_1 and NEAT1_2 compared with the uninfected cells. A recent study demonstrated that NEAT1 could affect the expression of cytokines in macrophages via the JNK/ERK MAPK pathway [13], which was involved in the upregulation of TNF-*α* and IL-6 during inflammatory responses. When the body is infected with Mtb, macrophages are activated to eliminate Mtb, prevent its spread, and increase the release of IL-6 to enhance the bactericidal ability of the body. Hence, the change in the levels of IL-6 after NEAT1_1 and NEAT1_2 silencing was detected in this study, and the result indicated that the secretion of IL-6 significantly declined after the knockout of NEAT1_1 and NEAT1_2 by siRNA, but there was no difference in IL-6 between NEAT1_1 and NEAT1_2 group. Counting of intracellular bacteria also showed that NEAT1_1 and NEAT1_2 knockout markedly weakened the clearance of Mtb by THP-1 cells, further confirming the involvement of NEAT1_1 and NEAT1_2 in post-Mtb immunity.

Indeed, this study had some limitations. For example, the expression of NEAT1_1 and NEAT1_2 in PBMCs of patients with latent tuberculosis was not detected, and therefore the role of NEAT1_1 and NEAT1_2 in latent tuberculosis remained unclear. This should be analyzed in further studies, and the sample size should be increased to make the results more reliable.

In conclusion, the present study reported that tuberculosis patients had significantly increased the expression of NEAT1 in their PBMCs, and the dynamic change in NEAT1 could reflect the efficacy of antituberculosis treatment. Therefore, NEAT1 might serve as a potential indicator for patient prognosis of tuberculosis. In addition, Mtb infection could increase the level of NEAT1 and further enhance continuous infection by promoting the release of inflammatory factors via multiple signaling pathways. Therefore, understanding the biological function and molecular mechanism of NEAT1 in tuberculosis would help provide new targets for the diagnosis or treatment of tuberculosis.

## Data Availability

The data used to support the findings of this study are included within the article.
